# Single-Incision Laparoscopic-Assisted Segmental Small-Bowel Resection for Small-Bowel Obstruction Caused by a Persimmon Bezoar: A Case Report

**DOI:** 10.70352/scrj.cr.26-0357

**Published:** 2026-06-24

**Authors:** Tsunehisa Kano, Kazuhiro Takahashi, Masahiko Hashimoto

**Affiliations:** Department of Surgery, Chigasaki Tokushukai Hospital, Chigasaki, Kanagawa, Japan

**Keywords:** bezoar, small-bowel obstruction, single-incision laparoscopic surgery, persimmon, phytobezoar

## Abstract

**INTRODUCTION:**

Small-bowel obstruction (SBO) caused by a bezoar is uncommon. We report a case of SBO due to a persimmon phytobezoar (diospyrobezoar) in a patient with a history of distal gastrectomy, treated by single-incision laparoscopic-assisted surgery (SILS) with segmental small-bowel resection.

**CASE PRESENTATION:**

An 83-year-old woman with a history of distal gastrectomy (Billroth I reconstruction) and habitual consumption of dried persimmons presented with abdominal pain and vomiting. Initial non-contrast CT showed small-bowel dilatation without a definitive intraluminal mass. A subsequent ileus-tube contrast study demonstrated a 50-mm oval filling defect with complete obstruction. Ultrasonography revealed an intraluminal mass with heterogeneous echoes and posterior acoustic shadowing, and repeat non-contrast CT identified a gas-containing intraluminal mass consistent with a bezoar. After adequate decompression via an ileus tube, SILS was performed on day 6 of admission. Intraoperatively, the bezoar was firmly impacted and immobile; therefore, segmental small-bowel resection with functional end-to-end anastomosis was performed. Histopathology showed submucosal edema, congestion, hemorrhage, and inflammatory cell infiltration, indicating pressure-related circulatory impairment.

**CONCLUSIONS:**

Firmly impacted persimmon bezoars may cause microscopic bowel-wall injury even without macroscopic necrosis. Segmental small-bowel resection can be a reasonable surgical option when bezoar impaction is severe and prolonged. SILS is a feasible minimally invasive approach in selected cases following adequate decompression.

## Abbreviations

SBO
: small-bowel obstruction
SILS
: single-incision laparoscopic-assisted surgery

## INTRODUCTION

Gastrointestinal bezoars are concretions of poorly digested material that form within the gastrointestinal tract. When a bezoar migrates into the small bowel, it can become impacted and cause mechanical obstruction. Bezoar-induced SBO accounts for approximately 0.4%–4% of all mechanical SBOs and is considered a relatively uncommon condition.^1)^ While chemical dissolution and endoscopic fragmentation may be options for gastric bezoars, surgical intervention is frequently required for bezoars impacted in the distal small bowel.^2)^ Persimmon bezoars (diospyrobezoars), in particular, are known to be harder and more resistant to dissolution than other phytobezoars; the reported initial success rate of cola dissolution therapy for persimmon bezoars is substantially lower than for other phytobezoars (23% vs. 60.6%, P = 0.022),^3)^ making treatment decisions more challenging. Herein, we report a case of SBO caused by a firmly impacted persimmon bezoar presenting 7 days after symptom onset, which was treated by SILS with segmental small-bowel resection. Histopathological examination revealed bowel-wall injury, even in the absence of macroscopic necrosis.

## CASE PRESENTATION

An 83-year-old woman presented with epigastric pain and vomiting. Her medical history included distal gastrectomy with Billroth I reconstruction approximately 40 years earlier (the precise indication was unclear due to the patient’s imprecise recollection), right femoral head replacement, atrial fibrillation (post-ablation), hypertension, aortic stenosis, and osteoporosis. She had a longstanding habit of consuming dried persimmons. Abdominal CT on admission demonstrated small-bowel dilatation; however, no definitive intraluminal mass or foreign body was identified (**Fig. 1**). She was admitted with a diagnosis of SBO.

**Fig. 1:**
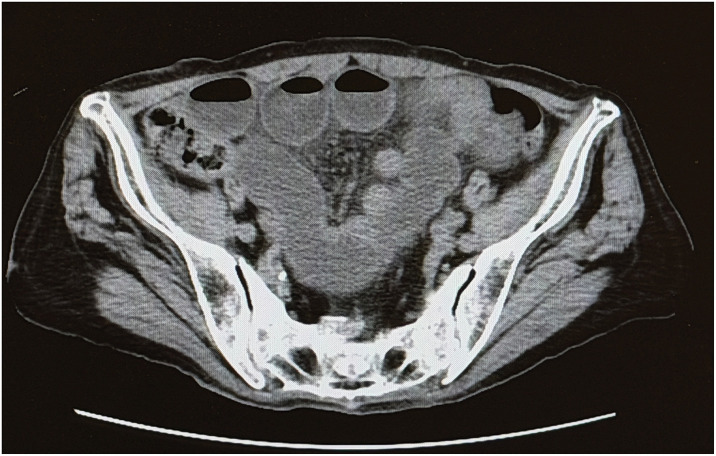
Abdominal non-contrast CT on admission. SBO is evident; however, the causative intraluminal mass cannot be identified.

SBO, small-bowel obstruction

On physical examination, a midline surgical scar was present on the upper abdomen. By the time of presentation, abdominal pain had partially resolved. The abdomen was flat and soft, with no tenderness or signs of peritoneal irritation. Laboratory findings included: white blood cells 4500/µL, red blood cells 352 × 10^4^/µL, hemoglobin 10.5 g/dL, C-reactive protein 3.81 mg/dL, aspartate aminotransferase 28 U/L, alanine aminotransferase 10 U/L, sodium 135 mmol/L, potassium 5.1 mmol/L, chloride 95 mmol/L, blood urea nitrogen 65.9 mg/dL, and creatinine 1.28 mg/dL.

Initial decompression was begun with a nasogastric tube. Subsequent history-taking revealed the patient’s prior gastrectomy and habitual dried-persimmon consumption, raising suspicion for a dietary bezoar as the cause of SBO. On day 3 of admission, the nasogastric tube was replaced with a long intestinal (ileus) tube for small-bowel decompression. Contrast study through the residual stomach via the ileus tube showed no gastric bezoar.

On day 5, an ileus-tube contrast study (**Fig. 2**) demonstrated an approximately 50-mm oval filling defect approximately 10–15 cm distal to the tube tip, with complete contrast obstruction at that level. Manual compression did not alter the shape of the defect, and contrast did not pass distally. Abdominal ultrasonography (**Fig. 3**) revealed a 57 × 28 × 53-mm oval intraluminal mass in the right lower abdomen, with well-defined borders, heterogeneous internal echoes, and posterior acoustic shadowing; the small bowel proximal to the mass was dilated. Repeat non-contrast CT (**Fig. 4**) identified a 45 × 31-mm intraluminal mass containing trace amounts of gas, distal to the ileus-tube tip. Based on these findings and the patient’s history of dried-persimmon consumption, SBO due to a persimmon bezoar was diagnosed.

**Fig. 2:**
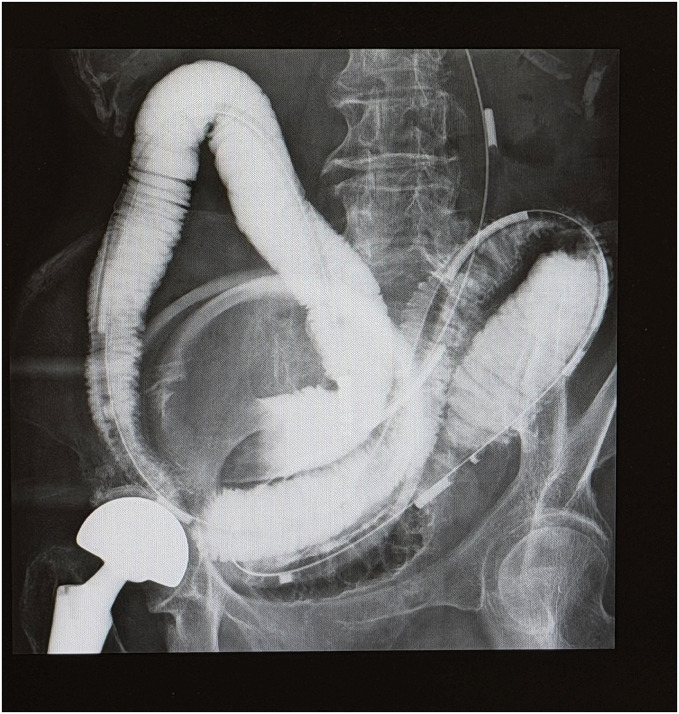
Ileus-tube contrast study. An oval intraluminal filling defect is identified, with complete obstruction at that site.

**Fig. 3:**
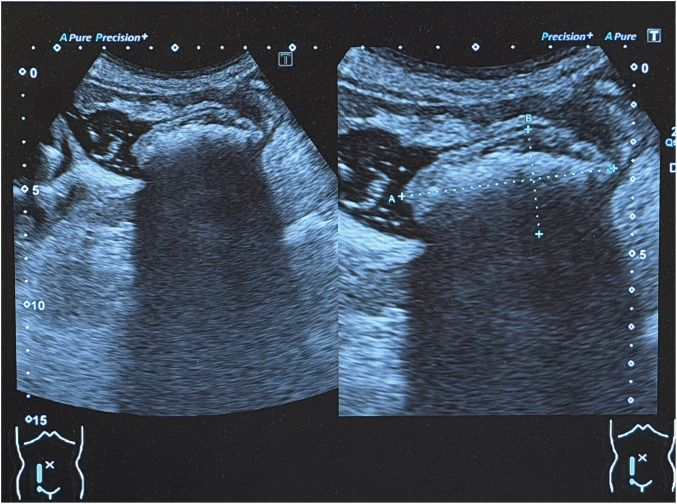
Abdominal ultrasonography performed after the ileus-tube contrast study. An intraluminal mass with posterior acoustic shadowing is identified distal to the dilated small bowel.

**Fig. 4:**
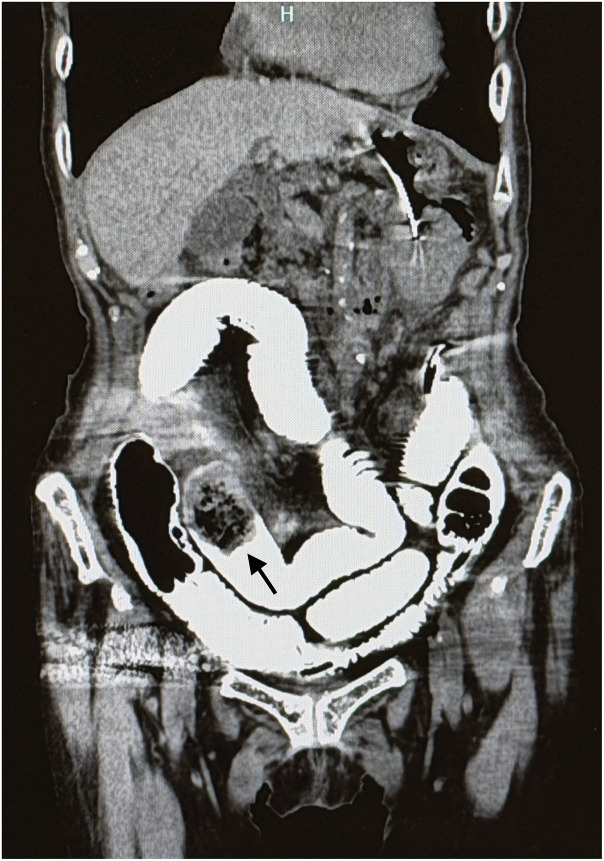
Repeat abdominal non-contrast CT. An intraluminal mass containing trace internal gas is clearly visualized distal to the ileus-tube tip (black arrow).

After adequate small-bowel decompression with resolution of intestinal dilatation, laparoscopic surgery was performed on day 6 of admission. A single approximately 3-cm incision was made just below the umbilicus, and SILS was initiated. Adhesions of the transverse colon to the abdominal wall were found in the upper abdomen as a result of prior surgery; however, no significant adhesions were noted in the lower abdomen. A focally dilated small-bowel loop caused by the impacted bezoar was readily identified. The affected bowel segment was exteriorized through the umbilical incision. Although the serosa showed no signs of serositis such as wall thickening, erythema, or edema, and no signs of circulatory impairment such as absent peristalsis or atonic bowel wall, the bezoar was firmly impacted and immobile, suggesting that chronic compressive mucosal injury could not be excluded; therefore, segmental small-bowel resection was performed, followed by functional end-to-end anastomosis.

Gross examination of the resected bowel showed a dark discoloration on the mucosal surface at the center of the specimen, without evident erosion or ulceration (**Fig. 5**). A hard, green-brown calculus was found within the resected segment. Infrared spectroscopy did not yield a definitive compositional identification; however, a spectrum similar to that of tannin was detected, and in conjunction with the patient’s dietary history, a persimmon bezoar was strongly suspected.

**Fig. 5:**
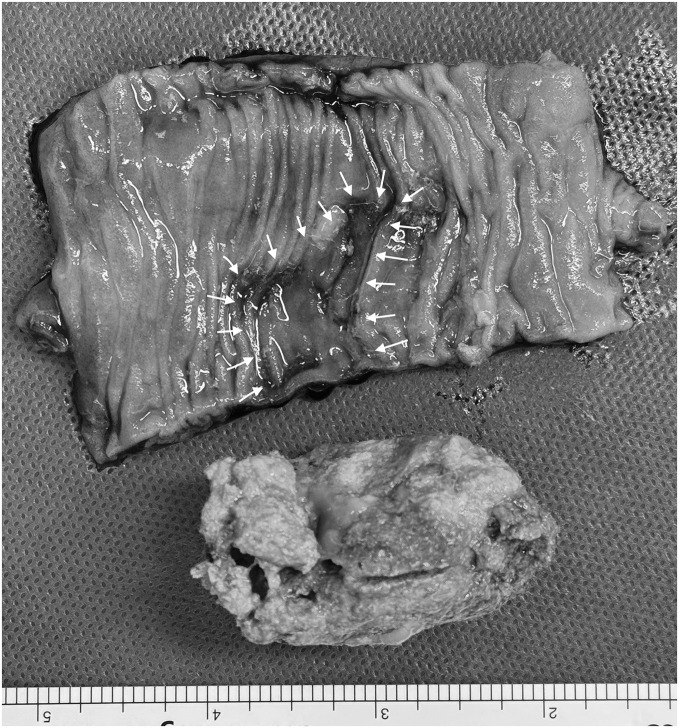
Gross appearance of the resected small-bowel segment and bezoar. The mucosal surface shows a well-demarcated discoloration with loss of Kerckring’s folds at the site of bezoar contact (white arrows), with the area of discoloration and fold loss sharing an identical sharp border, without evident erosion or ulceration.

Histopathological examination showed preservation of mucosal architecture without notable mucosal atrophy or desquamation; however, submucosal edema, congestion, hemorrhage, and inflammatory cell infiltration were observed, consistent with pressure-related circulatory impairment caused by bezoar compression.

The postoperative course was uneventful. Oral intake was resumed on POD 3, and the patient was discharged on POD 10. Because neither the patient nor her family could clearly recall the indication for the prior gastrectomy, dietary counseling was provided, including instructions to avoid high-fiber foods and to chew food thoroughly.

## DISCUSSION

Bezoar-induced SBO is relatively uncommon, and prior gastric surgery is a recognized predisposing factor.^4,5)^ Although abdominal CT is generally useful for diagnosis, definitive preoperative identification of the causative mass can be difficult in some cases.^6)^ In the present case, the initial non-contrast CT did not reveal an intraluminal mass. However, the history of gastrectomy raised suspicion of bezoar-induced SBO. Subsequent evaluation incorporating the dietary history of persimmon consumption, ileus-tube contrast study, ultrasonography, and repeat CT enabled a definitive preoperative diagnosis. Distal gastrectomy predisposes patients to bezoar formation through several mechanisms: reduced gastric acidity, impaired gastric motility, and altered gastric emptying due to loss of pyloric function collectively reduce the gastric processing of ingested material.^7)^ In the case of persimmon bezoars, these alterations promote the polymerization of persimmon tannins (shibuol) in an environment of reduced acidity, leading to the formation of an insoluble gelatinous mass that progressively hardens into a bezoar.^7)^ The present case is consistent with this mechanism, as the patient had undergone distal gastrectomy with Billroth I reconstruction and had a longstanding habit of consuming dried persimmons, a food with particularly high tannin content.

In terms of imaging diagnosis, CT has been reported to detect bezoars in all examined cases when specifically sought, whereas ultrasonography identified them in 15 of 17 patients and plain radiography in only 3 of 17.^8)^ However, these figures reflect series in which bezoar was already clinically suspected. As demonstrated in the present case, when the initial CT is performed without suspicion of bezoar, the intraluminal mass may be overlooked.^6)^ Ultrasonography, which can demonstrate a hyperechoic intraluminal mass with posterior acoustic shadowing, and ileus-tube contrast studies, which allow direct visualization of a filling defect at the obstruction site, serve as valuable adjuncts in such situations. A systematic diagnostic approach integrating dietary history, repeat imaging, and contrast studies is therefore recommended when bezoar-induced SBO is suspected but not confirmed on initial CT.^7)^

Regarding treatment, chemical dissolution and endoscopic interventions may be effective for gastric bezoars; however, persimmon bezoars are known to be more refractory to dissolution than other phytobezoars.^3)^ For bezoars impacted in the distal small bowel with resultant obstruction, surgical intervention remains the mainstay of treatment, with intraoperative options including milking the bezoar into the cecum or enterotomy for extraction.^2)^

In the present case, no macroscopic signs of bowel-wall compromise such as serositis or circulatory impairment were identified at the impaction site; nevertheless, segmental small-bowel resection was performed based on the firm impaction and complete immobility of the bezoar. On gross examination of the resected specimen, the area of mucosal discoloration and loss of Kerckring’s folds at the impaction site shared an identical well-demarcated border. This sharp demarcation is consistent with the clear-bordered mucosal injury pattern previously described in association with gastric bezoars^9)^ and may reflect the localized nature of pressure-related ischemic injury confined to the direct contact zone of the impacted bezoar. Histopathological analysis further demonstrated submucosal hemorrhage, congestion, and inflammatory cell infiltration, indicating pressure-related circulatory impairment that was not apparent on macroscopic inspection. A previously reported case of bezoar-induced SBO treated by enterotomy and extraction alone was complicated by a prolonged postoperative febrile course,^10)^ raising the possibility that occult mural injury at the impaction site may contribute to postoperative complications even when gross necrosis is absent. The histopathological findings of the present case support this hypothesis and highlight the potential limitations of relying solely on macroscopic assessment when deciding between enterotomy with extraction and segmental resection. These findings suggest that occult bowel-wall injury should be taken into account in surgical decision-making, particularly when the bezoar is firmly impacted and immobile.

Adequate preoperative decompression via the ileus tube led to the resolution of small-bowel dilatation, facilitating the minimally invasive procedure and allowing SILS to be completed with minimal intra-abdominal manipulation. SILS for SBO has been reported to be feasible in selected cases^11)^ and represents a viable minimally invasive option when patient selection is appropriate.

As this is a single case report, further accumulation of cases is needed to better clarify the relationship between histopathological changes at the bezoar impaction site and the optimal surgical procedure.

## CONCLUSIONS

SBO caused by an impacted persimmon bezoar was safely treated by SILS with segmental small-bowel resection following adequate preoperative decompression. In cases of firmly impacted bezoars with limited mobility, histopathological bowel-wall injury due to pressure-related circulatory impairment may be present even without macroscopic necrosis. Segmental small-bowel resection should therefore be considered as one of the surgical options in such situations.

## DECLARATIONS

### Use of artificial intelligence tools

Artificial intelligence tools (Claude, Anthropic) were used to assist with English translation and manuscript editing. The authors reviewed all content and take full responsibility for the accuracy and integrity of the manuscript.

### Availability of data and materials

The datasets supporting the conclusions of this article are included within the article.
